# Unemployment and Health

**Published:** 1922-09

**Authors:** 


### UNEMPLOYMENT AND HEALTH.

SIR MONTAGUE BARLOW, Parliamentary Secretary to the Minister of Labour, recently received a deputation from the People's League of Health on "The Effect of Unemployment and the Unemployment Insurance Benefit on the Health and Habits of the Nation." The deputation was introduced by Sir Alfred Fripp, K.C.V.O., C.B., M.S., F.R.C.S., and was composed of the following members of the Medical and Lay Councils of the League :—Dr. C. J. Bond, C.M.G., F.R.C.S., Chairman, Consultative Committee, Ministry of Health; Sir Bruce Bruce-Porter, K.B.E., C.M.G., M.D.; Col. S. Lyle Cummins, C.B., C.M.G., LL.D., M.D., Professor of Tuberculosis, South Wales University; Lt.-Col. Fremantle, M.P., M.A., F.R.C.P., D.P.H.; Dr. W. A. Potts, Psychological Expert to the Birmingham Justices; Capt. Donald Simson (representing the British Legion), and Miss Olga Nethersole, R.R.C.

Sir Bruce Bruce-Porter said that the amount paid by way of unemployment benefit, etc., was insufficient to keep the worker fit. In addition, he was frequently unversed in food values, and would be better able to render good service when trade revival came if he were able to obtain a standard balanced diet by means of food tickets in part substitution for unemployment benefit.

Col. Lyle Cummins found that whenever cessation of work occurred through bad trade, strikes, lock-outs, etc., the number of persons applying for treatment for tuberculosis soon increased, showing a direct communication between little or reduced income and ill health. He was asking the Ministry of Labour to consider the deterioration of workers in the country through the small means which were at their disposal through the unemployment benefit.

Dr. Potts said it was his experience with agitators, revolutionaries and delinquents that psychological disorders, frequently producing undesirable members of the community, could be traced to a considerable extent to ill health caused by insufficient food due to lack of remunerative work. He desired to emphasise that a change of occupation frequently had beneficial results, and therefore too much importance could not be attached to the plea often put forward that work could not be provided because the man's own trade was slack.

Dr. Bond spoke enthusiastically of the measures taken by the Leicester Distress Committee to institute public works, such as recreation grounds, road widening and general cleaning up. The steps taken in that city had proved beneficial, and the work was of a high order. The secret of success lay in the fact that unskilled labour had been diluted with a proportion of highly skilled workmen.

Capt. Simson, who had been nominated by Field-Marshal Lord Haig to speak on this subject for the British Legion, then referred to his scheme already before Sir Montague Barlow, by which the sums now spent on unemployment benefit might be diverted to a productive method of employment. Under this scheme benefit would be paid to local authorities and private employers in part payment of the wages of unemployed men taken on by them.

In reply, Sir Montague Barlow said that the Government were as anxious as anybody that work should be provided as far as possible. He fully appreciated the serious effect produced by prolonged unemployment on the health of the worker. As regards the suggestion made by Sir Bruce Bruce-Porter, there was a danger of improper use of the proposed food tickets which could be avoided only by elaborate supervisory machinery. With regard to Capt. Simson's scheme, it had to be remembered that the Insurance Fund was made up largely of the contributions of workpeople still in employment. They had contracted under the scheme for the payment to them of benefit when unemployed, and, as trustees of the fund, the Government could not divert the sums in their charge to purposes other than those provided under the scheme, except by legislation. Schemes on the lines of Capt. Simson's proposal were under consideration by the Government, though there were many difficulties in the way of their adoption.

